# Learning and depicting lobe-based radiomics feature for COPD Severity staging in low-dose CT images

**DOI:** 10.1186/s12890-024-03109-3

**Published:** 2024-06-24

**Authors:** Meng Zhao, Yanan Wu, Yifu Li, Xiaoyu Zhang, Shuyue Xia, Jiaxuan Xu, Rongchang Chen, Zhenyu Liang, Shouliang Qi

**Affiliations:** 1 College of Medicine and Biological Information Engineering, Northeastern University, Shenyang, China; 2 Key Laboratory of Intelligent Computing in Medical Image, Ministry of Education, Northeastern University, Shenyang, China; 3 Respiratory Department, Central Hospital Affiliated to Shenyang Medical College, Shenyang, China; 4 State Key Laboratory of Respiratory Disease, National Clinical Research Center for Respiratory Disease, Guangzhou Institute of Respiratory Health, The National Center for Respiratory Medicine, The First Affiliated Hospital of Guangzhou Medical University, Guangzhou, China; 5 Key Laboratory of Respiratory Disease of Shenzhen, Shenzhen Institute of Respiratory Disease, Shenzhen People’s Hospital (Second Affiliated Hospital of Jinan University, First Affiliated Hospital of South University of Science and Technology of China), Shenzhen, China

**Keywords:** Chronic obstructive pulmonary disease, Pulmonary lobe, Radiomics, Severity staging, Computed tomography

## Abstract

**Background:**

Chronic obstructive pulmonary disease (COPD) is a prevalent and debilitating respiratory condition that imposes a significant healthcare burden worldwide. Accurate staging of COPD severity is crucial for patient management and treatment planning.

**Methods:**

The retrospective study included 530 hospital patients. A lobe-based radiomics method was proposed to classify COPD severity using computed tomography (CT) images. First, we segmented the lung lobes with a convolutional neural network model. Secondly, the radiomic features of each lung lobe are extracted from CT images, the features of the five lung lobes are merged, and the selection of features is accomplished through the utilization of a variance threshold, t-Test, least absolute shrinkage and selection operator (LASSO). Finally, the COPD severity was classified by a support vector machine (SVM) classifier.

**Results:**

104 features were selected for staging COPD according to the Global initiative for chronic Obstructive Lung Disease (GOLD). The SVM classifier showed remarkable performance with an accuracy of 0.63. Moreover, an additional set of 132 features were selected to distinguish between milder (GOLD I + GOLD II) and more severe instances (GOLD III + GOLD IV) of COPD. The accuracy for SVM stood at 0.87.

**Conclusions:**

The proposed method proved that the novel lobe-based radiomics method can significantly contribute to the refinement of COPD severity staging. By combining radiomic features from each lung lobe, it can obtain a more comprehensive and rich set of features and better capture the CT radiomic features of the lung than simply observing the lung as a whole.

## Background

Chronic obstructive pulmonary disease (COPD) is a global public health challenge due to its widespread prevalence and its lasting impact on disability and mortality [1, 2]. COPD is currently ranked as the fourth most prevalent global health concern, marked by persistent airflow limitation and a range of debilitating symptoms [3].

Pulmonary function assessments, specifically the measurement of the ratio of forced expiratory volume in one second (FEV1) to forced vital capacity (FVC), serve as a primary diagnostic and risk assessment tool for COPD [4]. However, early COPD patients are easy to be ignored because of asymptomatic and mild symptoms [5, 6]. Most patients are often diagnosed with moderate to severe, which seriously affects the quality of life, and the cost of treatment has risen sharply [7]. Consequently, early identification and staging are important to reduce the risk of exacerbations, fewer concurrent health issues, and decreased healthcare expenses [8].

COPD is a multifaceted and remarkably diverse clinical condition with various imaging phenotypes and histopathological characteristics including encompassing parenchymal degradation, thickening of bronchial walls, interstitial lung abnormalities, bronchiectasis, and so on [9]. Computed tomography (CT)-based research in the field of COPD has yielded remarkable outcomes which has proven to be a powerful tool in studying COPD by providing detailed, three-dimensional images of the lungs [10–12]. CT imaging provides crucial insights into lung function, disease severity categorization, and the prediction of outcomes for individuals with COPD by examining typical CT features like lung tissue, airways, pulmonary blood vessels, and the chest wall. It has paved the way for more accurate diagnosis, personalized treatment strategies, and the development of innovative therapies, ultimately improving the lives of individuals living with COPD.

Radiomics was proposed by Lambin et al. in 2012 [13]. It entails the extraction and examination of numerous quantitative features, including texture, statistical, histogram, and shape features. [14]. Radiomics features in lung disease imaging have been considered cutting-edge tools for healthcare professionals [15]. Nevertheless, the evolution of radiomics features in COPD has been comparatively slower compared to other lung conditions like lung cancer and pulmonary nodules. As of 2020, Refaee et al. noted that there had been limited exploration of radiomics features in COPD [16]. Nevertheless, there are promising prospects for employing radiomics features in COPD diagnosis, treatment, and monitoring, as well as directions for future research [16]. Additionally, the significance of lung radiomics features in evaluating COPD has been substantiated [17].

Previous studies in radiomics for pulmonary conditions, specifically COPD, have primarily focused on analyzing radiomics features derived from the entire lung region. This study, however, overlooks the heterogeneity of COPD manifestations across different lung lobes. The severity of COPD and the distribution of lesions are known to vary considerably within the lung, often localized to specific lobes rather than uniformly affecting the entire lung region. Our study addresses this gap by proposing a lobe-specific radiomic analysis, allowing for a granular investigation into the distinct structural and functional characteristics of each lobe. The justification for the proposed method lies in the premise that individual lung lobes may contribute disparately to the pathology of COPD. Lesions within different lobes can exhibit unique radiomic signatures, which, when analyzed independently, provide more precise insights into the localized nature of the disease. This lobe-specific analysis enables a more accurate understanding of disease distribution and severity. By integrating radiomic features from each lobe, the proposed method seeks to offer a more nuanced understanding of COPD, pinpointing which lobes are most susceptible to the disease and the extent of their involvement.

Machine learning focuses on the development of algorithms and computer models. It learns from data and makes predictions without being explicitly programmed [18]. Statistical techniques are also involved to enable computers to automatically improve their performance on a specific task [19], for example, classification and staging of COPD [20, 21]. Yang et al. proposed to characterize and classify COPD stages based on multi-layer perceptron [22]. Makimoto and colleagues conducted a comparative analysis of various feature selection and classification methods, ultimately demonstrating that the combination of Elastic Net with a Linear-SVM classifier outperforms others for identifying COPD [23]. Puchakayala et al. demonstrated that radiomics features, particularly parenchymal texture, and shape features of the lung and airway, could accurately diagnose COPD in both standard-dose and low-dose CT images [24]. Support vector machine (SVM) is a supervised machine learning algorithm used for classification and regression tasks. It is particularly well-suited for classification problems by finding the optimal hyperplane. SVM is widely used in various fields which can be applied in diagnosing COPD and classifying its severity [25, 26].

We propose a novel lobe-based radiomic workflow and train a machine learning model for COPD severity staging. More specifically, five pulmonary lobes are segmented from lung CT images and radiomics features of each lobe are extracted, integrated, and dimension reduced. Then, an SVM classifier is employed to classify different severity of COPD, which has excellent performance. By combining radiomic features from each lung lobe, we can obtain a more comprehensive and rich set of features. This helps improve the performance of predictive models.

The contributions of the paper are as follows:

- Unlike the current radiomic features extracted from the whole lung region, this study introduces a novel approach by combining radiomics features from each lung lobe.
- The proposed workflow is effective in distinguishing COPD severity, exhibiting strong performance in both binary and multi-class classification tasks.
- By analyzing the contribution of each lung lobe separately, the study provides empirical evidence that COPD’s impact is localized, which enhances the understanding of the disease’s heterogeneity.

## Methods

### Datasets

The study received approval from the hospital’s ethics committee (Reference: ES-2023-045-01), and all participants gave informed consent in accordance with the Declaration of Helsinki (2000). COPD is diagnosed by evaluating the post-bronchodilator ratio of forced expiratory volume in one second (FEV1) to forced vital capacity (FVC), which is below 0.7 and further categorized into four stages based on the Global Initiative for Chronic Obstructive Lung Disease (GOLD) criteria: GOLD I (mild, FEV1 ≥ 80% predicted), GOLD II (moderate, FEV1 between 50% and < 80% predicted), GOLD III (severe, FEV1 between 30% and < 50% predicted), and GOLD IV (very severe, FEV1 < 30% predicted) [27].

Dataset 1, as a training dataset, collected 530 patients from the First Affiliated Hospital of Guangzhou Medical University. The number of patients in each stage is 114 in GOLD I, 204 in GOLD II, 154 in GOLD III, and 58 in GOLD IV. The excluded criteria are acute exacerbation of COPD or respiratory infection 4 weeks ago, concomitant pulmonary disease, pulmonary resection, and active malignancy within the past 5 years. Furthermore, Table 1 presents the clinical characteristics and parameter configurations for CT image acquisition.

**Table 1:** Baseline characteristics of dataset 1

Characteristics	Value
Age, yr, mean ± SD	65.5 ± 7.9
Sex, % female (n)	69.06 (530)
kVp, kV	117.41 ± 4.39
Slice thickness, mm, mean ± SD	0.99 ± 0.05
X-ray tube current, mA, mean ± SD	49.38 ± 21.78

Dataset 2 is from the Central Hospital Affiliated to Shenyang Medical College (CH-SMC) and the Second Hospital of Dalian Medical University (SH-DLMU) as an external validation dataset. Dataset 2 consists of 290 patients: 25 GOLD I, 69 GOLD II, 116 GOLD III, 70 GOLD IV. A summary of the stages of COPD patients in Dataset 2 is shown in Table 2.

**Table 2:** Summary of stages of COPD patients in the dataset 2

Hospital	GOLD I	GOLD II	GOLD III	GOLD IV	Total
CH-SMC	2	36	89	64	191
SH-DLMU	23	43	27	6	99
Total	25	79	116	70	290

### Overview of the study procedure

**Fig. 1:**
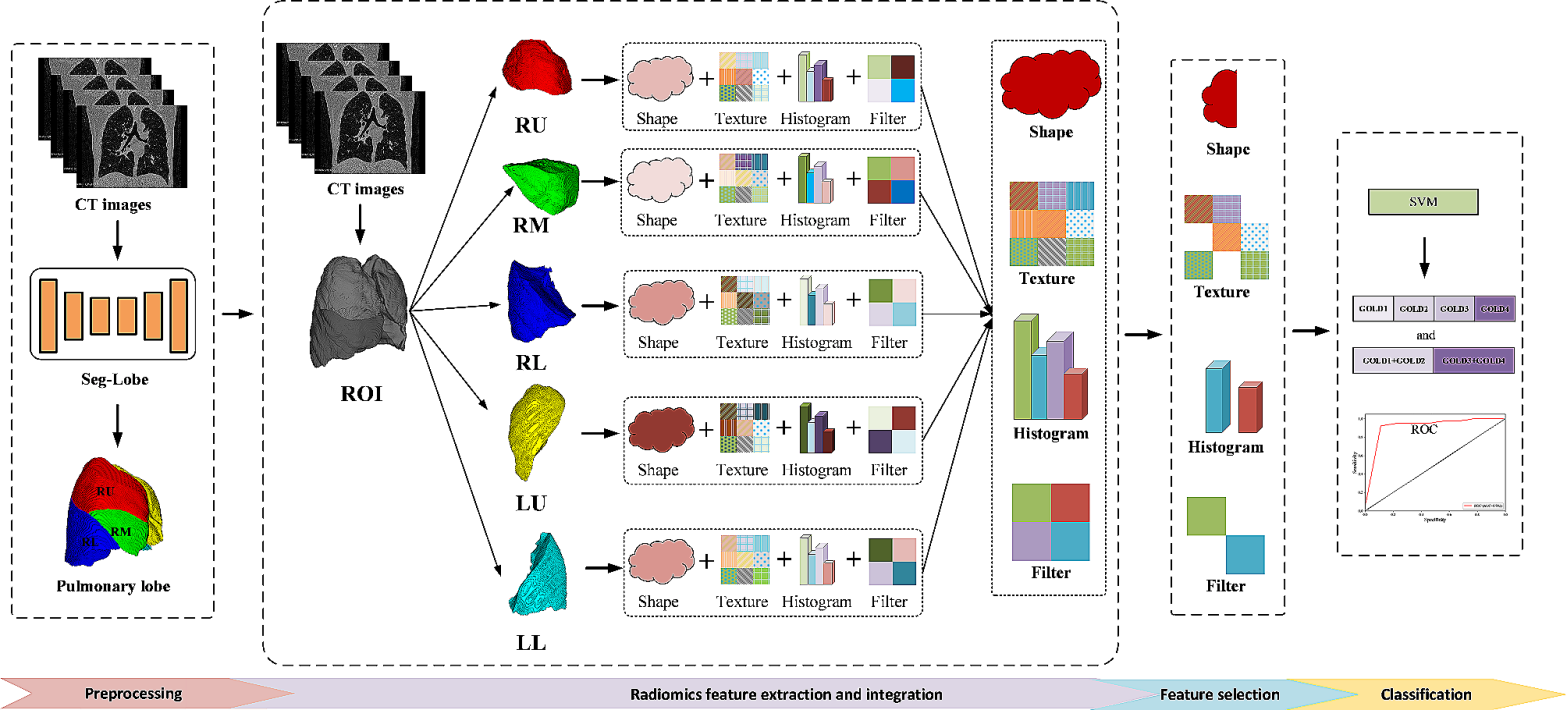
The lobe-based radiomics workflow for COPD stage classification

The pipeline of the proposed lobe-based method for this study is depicted in Fig. 1. First, five lobes are segmented from the lung CT images by the trained Seg-Lobe model, including the right upper (RU), right middle (RM), and right lower (RL) lobe in the right lung, left upper (LU) and the left lower (LL) lobe in the left lung. Second, radiomics features are extracted from regions of interest (ROI) in the lung (the region of five segmented lobes), respectively. And the radiomics features from each lobe are combined. Third, the integrated features are selected using the LASSO algorithm. Finally, an SVM classifier is built to classify the severity staging of COPD.

### Preprocessing

**Fig. 2:**
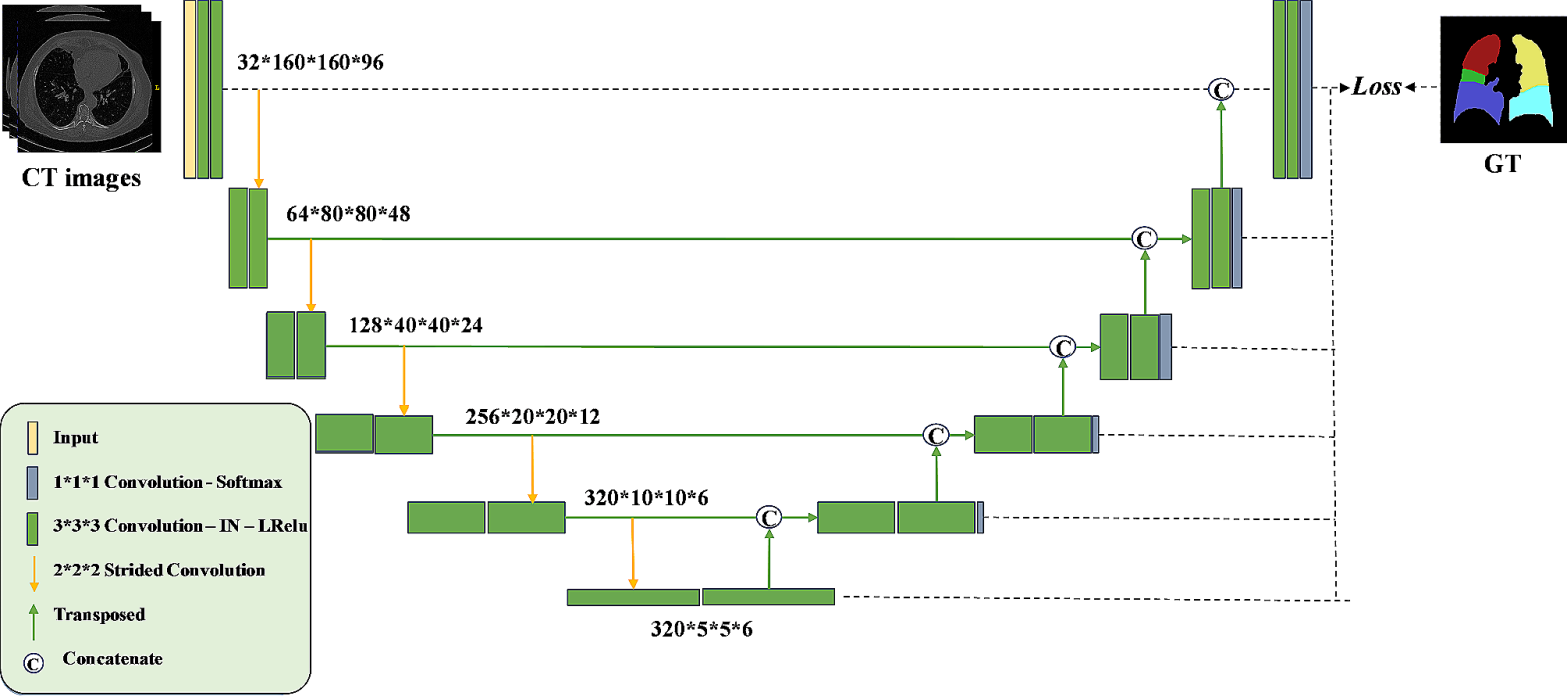
The architecture of the Seg-Lobe

The segmentation of lung lobes is performed by using the Seg-Lobe model, which is based on 3D U-Net [28] and implemented as the automatic pipeline to train, validate, and test the network using the input CT images [29, 30]. The details are provided in our previous work [27]. The Seg-Lobe architecture, illustrated in Fig. 2, comprises five encoder-decoder pairs. Each encoder or decoder is constructed with two blocks, each consisting of a sequence of operations: convolution, instance normalization (IN) [31], and Leaky Rectified Linear Unit (Leaky ReLU). Although Batch Normalization (BN) [32] is commonly used to enhance training speed and stability, its effectiveness diminishes when applied to small batch sizes.

In Seg-Lobe model architecture, strided convolution is performed instead of an ordinary pooling operation in downsampling, which serves to enhance the accuracy of the convolutional neural network while concurrently reducing the model’s size [33, 34]. For upsampling, transposed convolution is employed. Furthermore, a skip connection [35] is implemented between the encoder and decoder to establish a connection between feature maps. This connection allows the decoder to access the information concealed within the encoder, facilitating more effective information flow.

### Feature extraction

Pyradiomics [36] is employed for feature extraction from the region of interest in CT images. It is a crucial and advanced Python package in the field of medical image analysis. The extracted features can be divided into seven groups: (a) first order features, (b) shape features, (c) gray level co-occurrence matrix (GLCM), (d) gray level size zone matrix (GLSZM), (e) gray level run length matrix (GLRLM), (f) neighboring gray Tone difference matrix (NGTDM), and (g) gray level dependence matrix (GLDM). For comprehensive definitions and detailed explanations of these texture features, the Pyradiomics official documents should be consulted [37].

### Feature selection

Feature selection helps improve the performance of predictive models by identifying and retaining the most relevant and informative features, mitigates the risk of overfitting and reduces dimensionality by eliminating irrelevant or redundant variables. This study employs three methods for the gradual selection of optimal features.

In the feature selection of the four categories of COPD severity, the variance threshold is first used to identify and retain features that exhibit significant variation across the dataset. Features with low variance, implying minimal variation across data points, are often considered less informative. In this experiment, we set the threshold to 1 and features failing to meet this threshold are subsequently discarded. In the feature selection of the two categories of COPD severity, COPD groups are classified according to GOLD stage in groups of mild severity (GOLD I + GOLD II, FEV1 < 50%) and great severity (GOLD III + GOLD IV, FEV1 ≥ 50%). The T-test is used to evaluate whether there is a significant difference between the two categories. Features exhibiting a significant difference (*p* < 0.05) are retained as they are considered discriminatory.

Finally, the least absolute shrinkage and selection operator (LASSO) method was utilized for the identification of the ultimate discriminative features [38]. LASSO incorporates a regularization term into the linear regression objective function. This method can reduce the coefficients of variables with minimal impact on the regression to zero during the fitting process, thereby achieving variable screening and complexity adjustment [39, 40]. LASSO involves a tuning parameter responsible for controlling the penalty applied to the linear model. This parameter is designed to maintain a minimal penalty while deriving a model with a reduced set of features. In this context, the penalty is expressed through the mean square error (MSE). The optimization objective of LASSO is as follows:

1 $$\begin{array}{c}y=\left(\frac{1}{2*{n}_{samples}}\right)*{\left|\left|\gamma -X\omega \right|\right|}^{2}+\alpha *\left|\left|\omega \right|\right|\end{array}$$

In the equation, *X* represents the matrix of radiomics features, $\gamma$ is the sample vector marker, *n* denotes the sample number, $\omega$ is the coefficient vector of the regression model, and $\alpha *\left|\left|\omega \right|\right|$ represents the LASSO penalty term.

### Machine-learning classification model

In this study, SVM is used to establish four-category and two-category classification models. SVM, a non-probabilistic supervised learning method, excels at forming multi-dimensional hyperplanes that efficiently separate the covariate space into distinct groups for classification. To evaluate the reliability of our proposed approach, we implemented a 5-fold cross-validation procedure on the complete dataset. More precisely, we randomly divided the data into five groups, using each set of four groups for training and the remaining one for testing.

### Statistical analysis and experimental setup

The classification performance is evaluated by the accuracy (ACC), precision, recall, F1-score, receiver operating characteristic (ROC) curve, and area under the curve (AUC).

2 $$\begin{array}{c}Accuracy= \frac{{\sum }_{i=1}^{4}T{P}_{i}}{{\sum }_{i=1}^{4}{TP}_{i}+F{P}_{i}+F{N}_{i}+T{N}_{i}}\end{array}$$

3 $$\begin{array}{c}Precision=\frac{T{P}_{i}}{T{P}_{i}+F{P}_{i}}, i=1, 2, 3, 4.\end{array}$$

4 $$\begin{array}{c}Recall=\frac{T{P}_{i}}{T{P}_{i}+F{N}_{i}}, i=1, 2, 3, 4.\end{array}$$

5 $$\begin{array}{c}F1-score=2*\frac{\text{P}\text{r}\text{e}\text{c}\text{i}\text{s}\text{i}\text{o}\text{n}\text{*}\text{R}\text{e}\text{c}\text{a}\text{l}\text{l}}{\text{P}\text{r}\text{e}\text{c}\text{i}\text{s}\text{i}\text{o}\text{n}+\text{R}\text{e}\text{c}\text{a}\text{l}\text{l}}, i=1, 2, 3, 4.\end{array}$$

As described in our previous study [41], let $T=({T}_{1},{T}_{2},{T}_{3},{T}_{4},)$, i.e. represent the target labels in the test set. The predicted classes in the test set, denoted as $P=\left({P}_{1},{P}_{2},{P}_{3},{P}_{4},\right)$, are determined. True positive (TP) is defined as the count of predicted labels matching the target labels, i.e. $T{P}_{i}=\left|{P}_{i}\cap {T}_{i}\right|, i=1, 2, 3, 4$. False positive (FP) represents the count of predicted labels that do not match the actual target labels, i.e. $F{P}_{i}=\left|{P}_{i}\backslash {T}_{i}\right|, i=1, 2, 3, 4$. False negative (FN) is the count of predictions that belong to the ground-truth label but are falsely predicted, i.e. $F{N}_{i}=\left|{T}_{i}\backslash {P}_{i}\right|, i=1, 2, 3, 4$. True negative (TN) is the count of predictions that neither belong to the ground-truth label nor are classified, i.e. $T{N}_{i}=\sum _{d?C,d\ne c}|{T}_{d}|$.For the classification of two categories of COPD severity, the evaluation index is calculated similarly to the above process, with the adjustment i = 1, 2.

The ROC curve illustrates the association between false positive rate (FPR) on the x-axis and true positive rate (TPR) on the y-axis [42]. The area under the curve (AUC) is computed from the ROC curve and serves as a metric reflecting the performance of a classifier. A higher AUC value, nearing 1.0, indicates a more effective classifier [43].

In this study, the machine learning models, such as SVM, KNN, and Decision Tree, were primarily implemented using the scikit-learn library. For the implementation of more advanced ensemble methods, specifically gradient boosting techniques, we employed the XGBClassifier from the XGBoost package and the CatBoostClassifier from the CatBoost package. Both of these classifiers were utilized with their default parameter settings. The use of default parameters also facilitates more straightforward comparisons between the different models and highlights the out-of-the-box capabilities of each algorithm.

## Results

### Radiomic feature selection in four and two categories of severity staging of COPD

In the lobe-combined experiment, 8840 radiomic features were extracted from a total of five lung lobes. Feature dimensionality reduction was carried out for two and four categories in the severity staging of COPD.

**Fig. 3:**
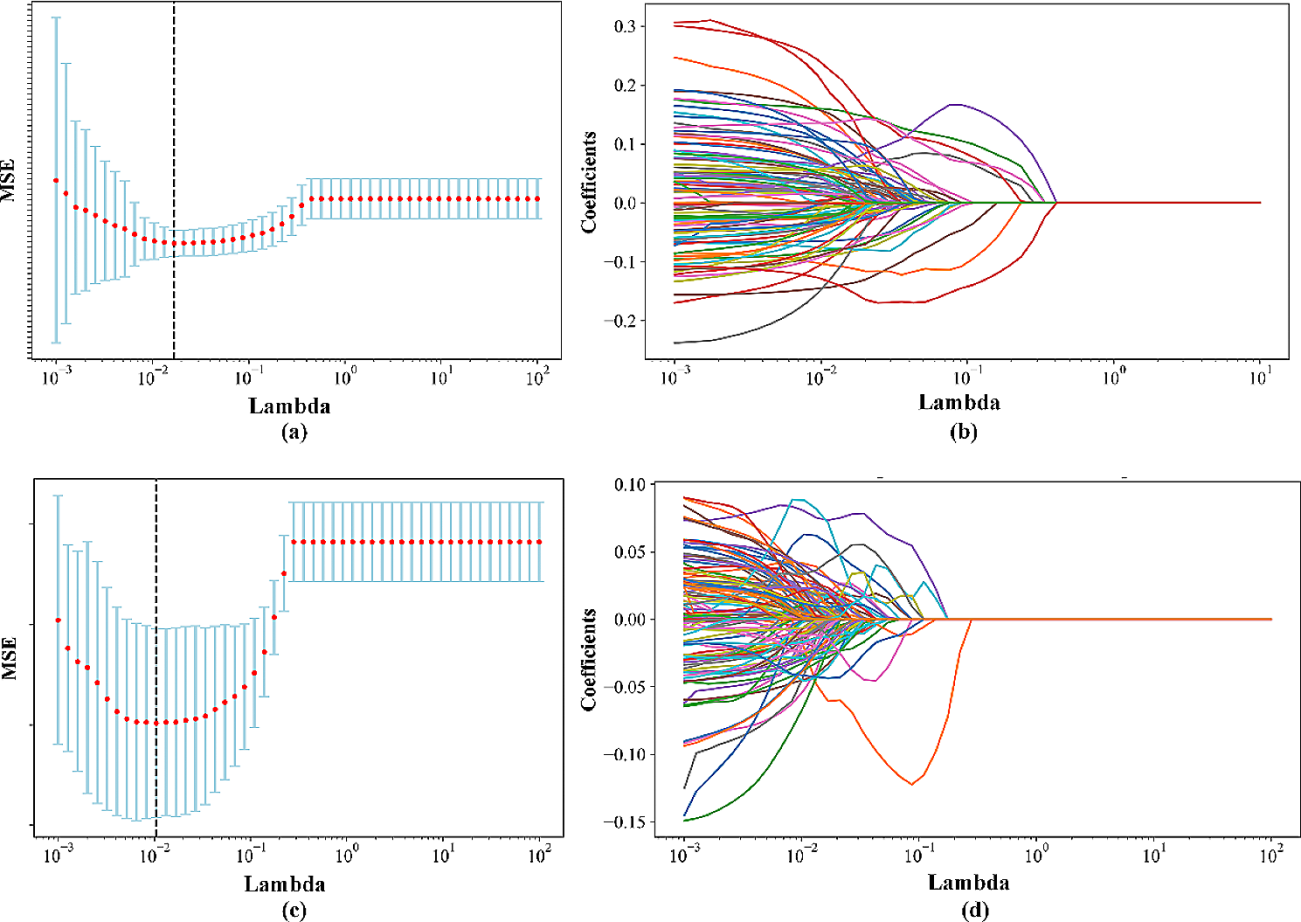
Changes in Mean Squared Error (MSE) and the coefficients of individual features concerning Lambda in the LASSO. (**a**) The MSE for the four categories. (**b**) The coefficient of each feature for the four categories. (**c**) The MSE for the two categories. (d) The coefficient of each feature for the two categories

In the four categories of COPD severity, 1370 features were selected by a variance threshold method. In Fig. 3(a), the MSE in LASSO is depicted concerning Lambda, while Fig. 3(b) illustrates the change in each feature coefficient corresponding to Lambda. As shown by the dotted line in Fig. 3(a), MSE attains its minimum value, resulting in the reduction of features to 104 through the LASSO algorithm.

**Fig. 4:**
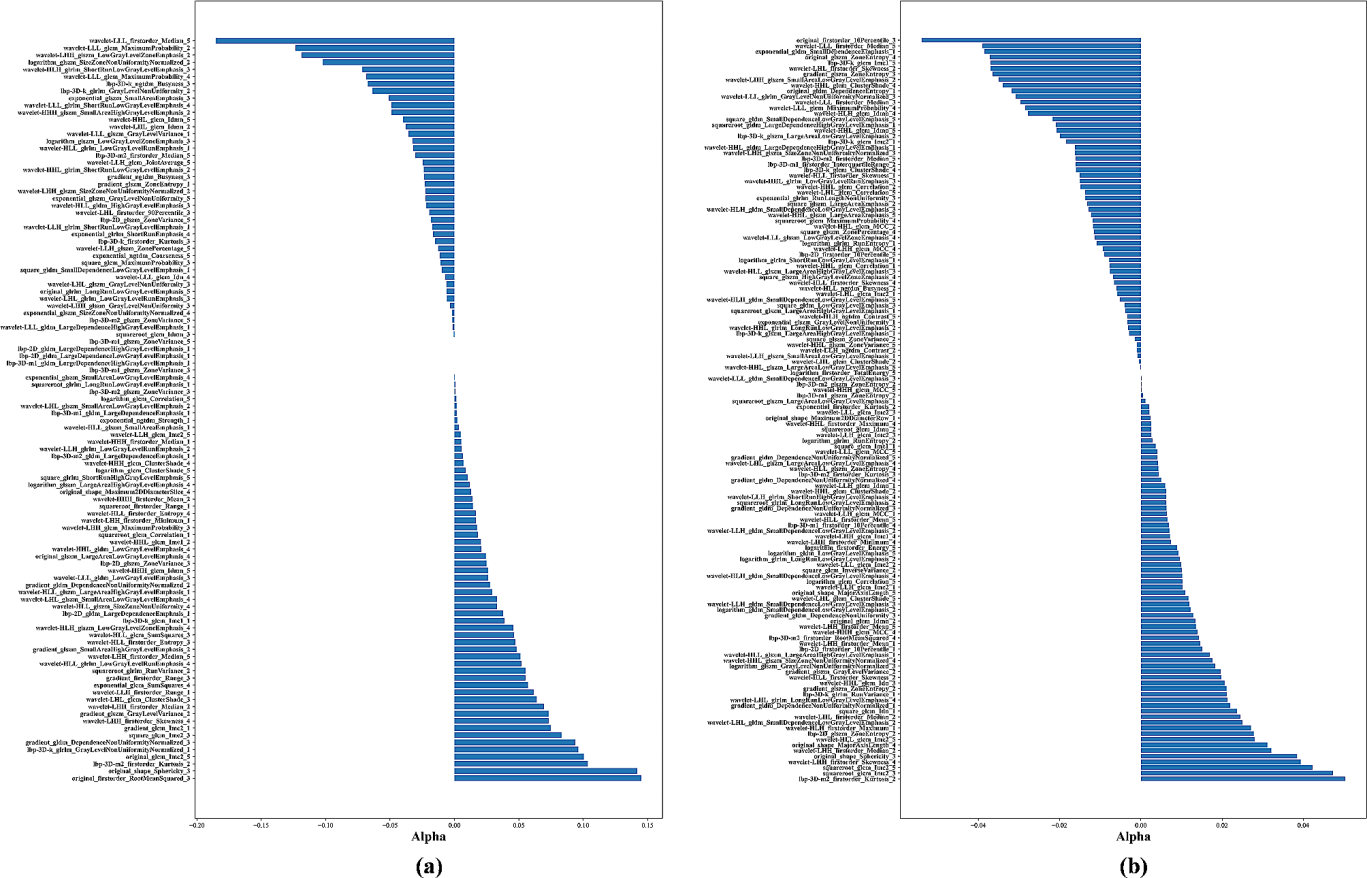
Optimal coefficients of features within LASSO for the staging of COPD severity. (**a**) The four categories. (**b**) The two categories

From the initial pool of 8840 features, 4764 features were chosen using the t-test method in the two categories. In Fig. 3(c), the MSE is depicted throughout the Lambda parameter optimization in the LASSO algorithm. Figure 3(d) illustrates that the LASSO algorithm selected 132 optimal features when the MSE reached its minimum, as denoted by the dotted line. Figure 4(a) presents the coefficients of the selected features in the LASSO model for the four categories, while Fig. 4(b) shows the coefficients of the selected features in the LASSO model for the two categories of COPD severity staging.

### Performance comparisons in four and two categories of severity staging of COPD with different feature selection methods

Table 3 presents the outcomes of various radiomics experiments applied to stage the severity of COPD. The SVM classifier is trained for both four and two categories of COPD severity staging, utilizing selected radiomics features from three distinct ROIs: the entire lung region, each lobe individually, and a combination of all five lobes.

We present the performance evaluation of lobe-based radiomics methods compared to other-region radiomics methods for staging COPD severity in Dataset 1. The task is categorized into four categories representing GOLD I vs. GOLD II vs. GOLD III vs. GOLD IV, and two categories representing GOLD I + GOLD II vs. GOLD III + GOLD IV. For the four-category classification task, the whole lung method achieves an accuracy of 0.49, with precision, recall, and F1-score all around 0.45. Among the lobe-based methods, RM (Right Middle) exhibits the highest performance metrics, with accuracy, precision, recall, and F1-score ranging from 0.55 to 0.67. Notably, our proposed method outperforms both the whole lung and lobe-based methods, achieving an accuracy of 0.63 and precision, recall, and F1-score all above 0.60, albeit with a slightly lower AUC of 0.49.

In the two-category classification task, the performance of all methods notably improves, with the proposed method consistently outperforming others. Specifically, the proposed method achieves an accuracy of 0.87, with precision, recall, and F1-score all at 0.87, and an AUC of 0.93, indicating its robustness and effectiveness in distinguishing between less severe and more severe COPD stages.

**Fig. 5:**
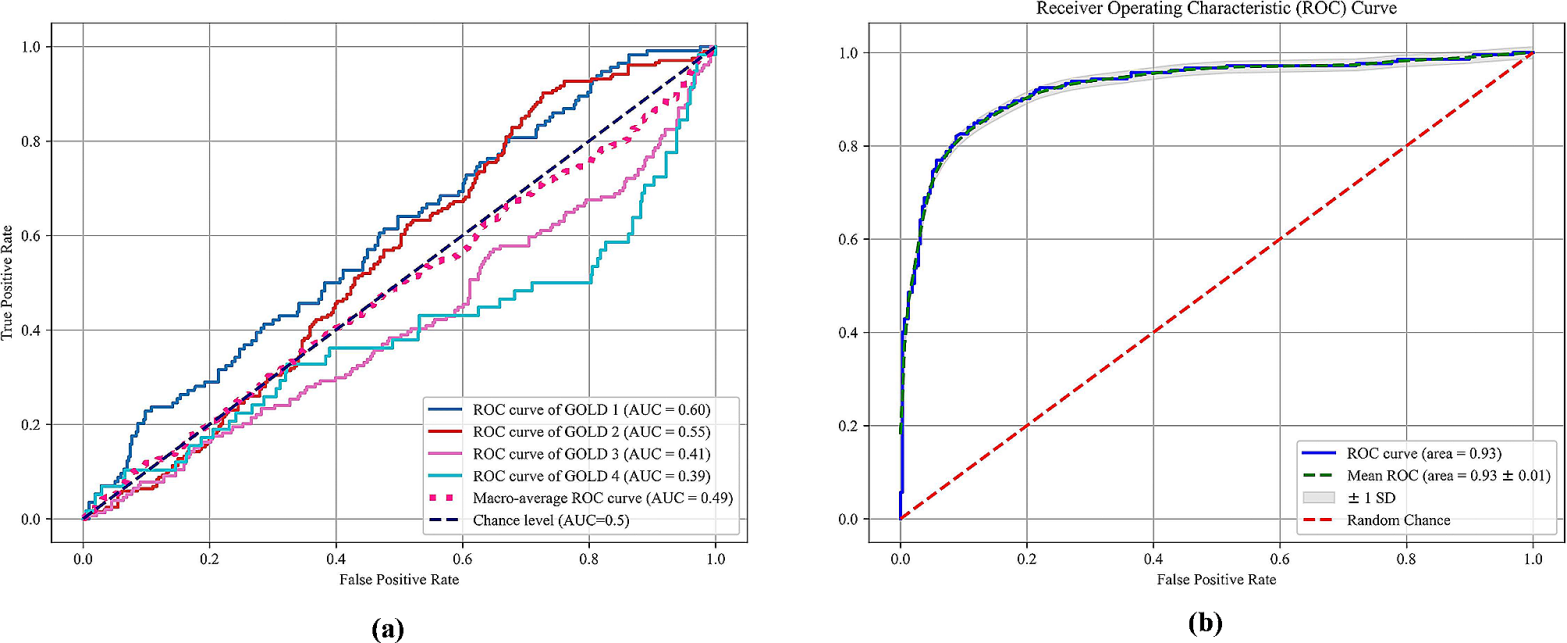
The ROC curves using SVM for COPD severity staging based on a combination of five lobes radiomic features. (**a**) Four categories; (**b**) Two categories

Overall, the results demonstrate the superiority of our proposed lobe-based radiomics method over other-region radiomics methods in accurately staging COPD severity, particularly evident in the two-category classification task, where it achieves the highest performance metrics across all evaluated criteria. The evaluation of two COPD severity classifications, based on selected radiomic features from all five lung lobes and analyzed using SVM, is depicted in Fig. 5(b) through ROC curve analysis.

The above experiments demonstrate that regardless of whether COPD severity is classified into four or two categories, the results of the radiomics model using combined features of five lung lobes are superior to that of using radiomic features directly from the whole lung region and each lung lobe. Overall, the results demonstrate the superiority of our proposed lobe-based radiomics method over other-region radiomics methods in accurately staging COPD severity, particularly evident in the two-category classification task, where it achieves the highest performance metrics across all evaluated criteria.

**Table 3:** Performance of lobe-based radiomics method compared with other-region radiomics method for COPD severity staging in dataset 1. Four categories represent GOLD I vs. GOLD II vs. GOLD III vs. GOLD IV and two categories represent GOLD I + GOLD II vs. GOLD III + vs. GOLD IV on Dataset1

Task	Radiomics method		Accuracy	Precision	Recall	F1-score	AUC
Four categories	Whole lung		0.49	0.45	0.49	0.45	0.46
	Lobe	RU	0.52	0.57	0.52	0.50	0.46
		RM	0.55	0.67	0.55	0.53	0.45
		RL	0.50	0.49	0.50	0.48	0.46
		LU	0.48	0.48	0.48	0.45	0.47
		LL	0.49	0.46	0.49	0.44	0.46
	**Proposed method**		**0.63**	**0.71**	**0.63**	**0.62**	**0.49**
Two categories	Whole lung		0.80	0.80	0.80	0.80	0.86
	Lobe	RU	0.78	0.78	0.78	0.77	0.84
		RM	0.78	0.78	0.78	0.78	0.86
		RL	0.82	0.82	0.82	0.81	0.87
		LU	0.78	0.78	0.78	0.77	0.84
		LL	0.81	0.81	0.81	0.81	0.86
	**Proposed method**		**0.87**	**0.87**	**0.87**	**0.87**	**0.93**

In Table 4, we present the performance comparison between the LASSO and PCA methods for COPD severity staging on Dataset 1, categorized into four categories (representing GOLD I vs. GOLD II vs. GOLD III vs. GOLD IV) and two categories (representing GOLD I + GOLD II vs. GOLD III + GOLD IV).

For the four-category classification task, the PCA method achieves an accuracy of 0.45, with precision, recall, and F1-score all around 0.41. In contrast, the LASSO method outperforms PCA significantly, with an accuracy of 0.63 and precision, recall, and F1-score all above 0.60.

In the two-category classification task, both PCA and LASSO methods demonstrate improved performance compared to the four-category classification. However, the LASSO method continues to exhibit superior performance, achieving an accuracy of 0.87 and precision, recall, and F1-score all at 0.87, whereas PCA achieves an accuracy of 0.65 with slightly lower precision, recall, and F1-score.

Overall, the results suggest that the LASSO method outperforms PCA in accurately staging COPD severity, particularly evident in the higher accuracy, precision, recall, and F1-score achieved across both classification tasks. This underscores the effectiveness of LASSO as a feature selection method for radiomics-based COPD severity staging on Dataset 1.

**Table 4:** Performance of LASSO compared with PCA method for COPD severity staging on Dataset1

Task	Feature selection	Accuracy	Precision	Recall	F1-score
Four categories	PCA	0.45	0.41	0.45	0.41
	**LASSO**	**0.63**	**0.71**	**0.63**	**0.62**
Two categories	PCA	0.65	0.64	0.65	0.63
	**LASSO**	**0.87**	**0.87**	**0.87**	**0.87**

### Performance comparisons in four and two categories of severity staging of COPD with different machine learning methods

In Table 5, we present a comprehensive evaluation of various machine learning classifiers for COPD severity staging on Dataset 1 (Training dataset). The task encompasses both four-category (GOLD I vs. GOLD II vs. GOLD III vs. GOLD IV) and two-category (GOLD I + GOLD II vs. GOLD III + GOLD IV) classification scenarios. For the four-category classification, classifiers including KNN, Decision Tree, AdaBoost, Gradient Boosting, XGBoost, Random Forest, CatBoost, and our proposed lobe-based radiomics method are examined. In the task of four-category classification, the proposed approach demonstrates superior performance compared to other classifiers, attaining an accuracy of 0.63, precision of 0.71, recall of 0.63, and F1 score of 0.62. The training time of the five classifiers is also provided in Table 5.

In the two-category classification task, the proposed method again shows superior performance, achieving perfect scores of 0.87 across all metrics. The runner-up, Gradient Boosting and CatBoost, scores 0.84 across all metrics.

Although the accuracy of our proposed method is slightly lower than XGBoost and CatBoost in the four-way classification task of COPD severity, it is still competitive, but it has a clear advantage in the two-way classification of COPD severity. These results demonstrate the effectiveness of the proposed method in staging COPD severity, providing strong evidence for its potential use in clinical settings.

**Table 5:** Performance of proposed lobe-based radiomics method compared with other machine learning methods for COPD severity staging on Dataset1

Task	Classifier	Accuracy	Precision	Recall	F1-score	AUC	Training time (s)
Four categories	KNN	0.50	0.53	0.50	0.49	0.47	--
	Decision Tree	0.52	0.57	0.52	0.50	0.48	0.0349
	AdaBoost	0.51	0.51	0.51	0.50	0.44	0.2813
	Gradient Boosting	0.57	0.57	0.57	0.56	0.45	4.6552
	XGBoost	0.64	0.66	0.64	0.60	0.45	0.5206
	Random Forest	0.60	0.68	0.60	0.59	0.44	0.3022
	CatBoost	0.64	0.68	0.64	0.63	0.48	150.8
	**Proposed method**	**0.63**	**0.71**	**0.63**	**0.62**	**0.49**	**0.0621**
Two categories	KNN	0.80	0.80	0.80	0.79	0.87	--
	Decision Tree	0.73	0.73	0.73	0.73	0.72	0.0312
	AdaBoost	0.79	0.79	0.79	0.79	0.85	0.3504
	Gradient Boosting	0.84	0.84	0.84	0.84	0.89	1.5213
	XGBoost	0.82	0.82	0.81	0.82	0.89	0.1366
	Random Forest	0.83	0.83	0.82	0.83	0.89	0.3191
	CatBoost	0.84	0.84	0.84	0.84	0.88	64.7
	**Proposed method**	**0.87**	**0.87**	**0.87**	**0.87**	**0.93**	**0.0440**

### Performance of testing on dataset 2

We extended the application of our methodology to an external dataset, Dataset 2 was tested on the previously trained model. Utilizing an SVM classifier, we categorized the features—post feature selection—into two categories of COPD severity.

As shown in Table 6, for the four-category classification task, the proposed method achieves an accuracy of 0.50, precision of 0.47, recall of 0.50, F1-score of 0.45, and AUC of 0.48. In the two-category classification task, the proposed method demonstrates improved performance, achieving an accuracy of 0.81, precision of 0.81, recall of 0.81, F1-score of 0.81, and AUC of 0.88. These results suggest that the proposed method performs well in distinguishing between less severe (GOLD I and II) and more severe (GOLD III and IV) COPD stages.

It is important to note that the training was conducted on Dataset 1, consisting of low-dose lung CT scans, whereas Dataset 2, employed for external validation, contained standard-dose CT images. This difference in dosing may account for the marginally lower performance observed on Dataset 2.

**Table 6:** The performance of the proposed method for COPD severity staging on dataset 2

Task	Accuracy	Precision	Recall	F1-score	AUC
Four categories	0.50	0.47	0.50	0.45	0.48
Two categories	0.81	0.81	0.81	0.81	0.88

### Depicting the importance of each lobe radiomics feature in COPD staging

**Fig. 6:**
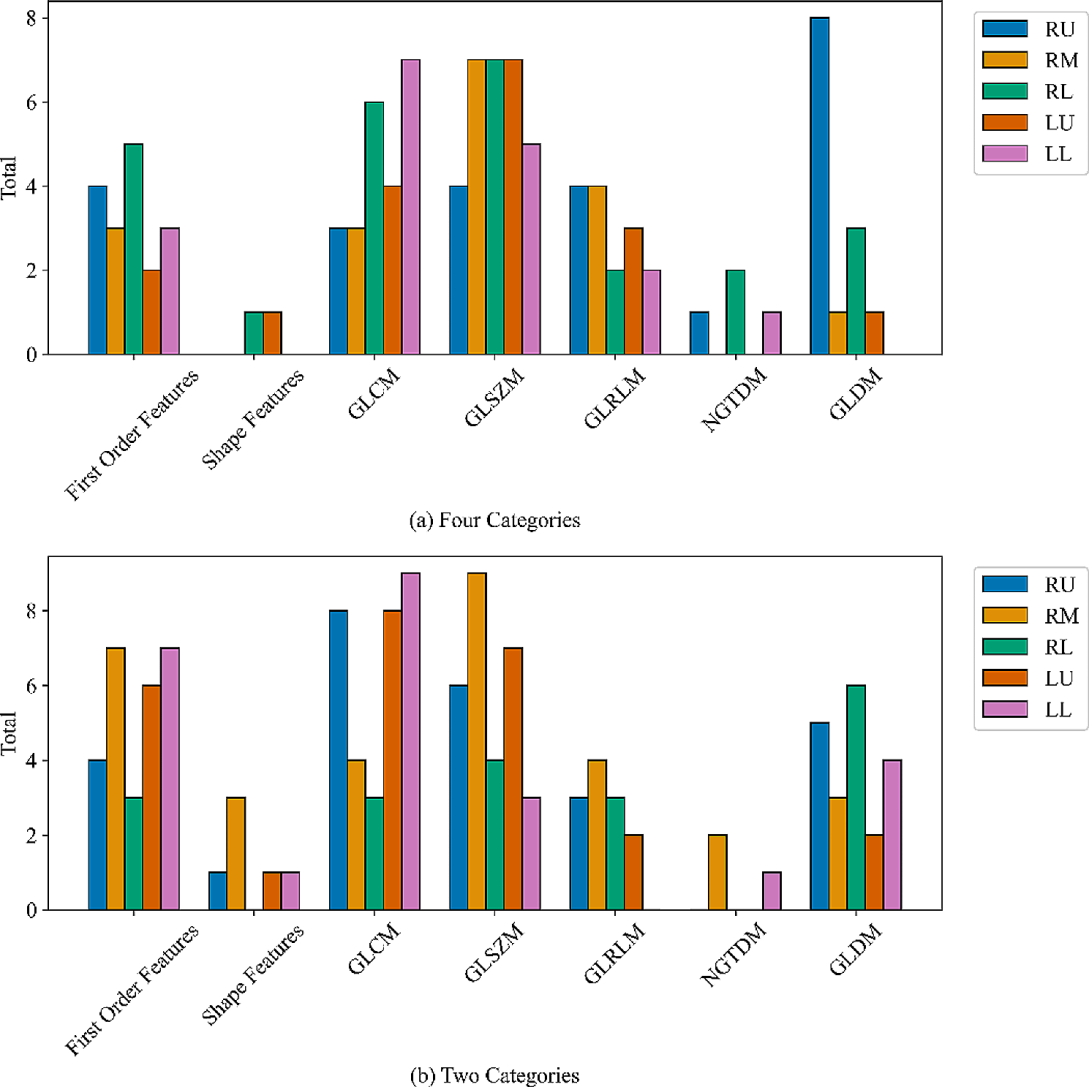
The feature species and the contribution of each lung lobe in feature selection of COPD severity. (**a**) Four categories; (**b**) Two categories

We analyze the importance of the radiomics feature in each lobe for COPD staging. Figure 6 shows the contribution of radiomics features of each lung lobe in the feature selection of four classifications of COPD severity. Seven types of features are collected, including first order features, shape features, GLCM, GLSZM, GLRLM, NGTDM, and GLDM.

In the four-category classification task. First Order Features are most prevalent in the RL lobe with 5 occurrences, totaling 17 across all lobes. Shape Features are relatively rare, with just 2 occurrences across all lobes and only appearing in the RM and LU lobes. GLCM features are most common in the LL lobe with 7 occurrences, with a total of 23 across all lobes. GLSZM features are evenly distributed across the RM, RL, and LU lobes with 7 occurrences each, totaling 30 across all lobes. GLRLM and GLDM features are most prevalent in the RU lobe, with 4 and 8 occurrences respectively.

In the two-category classification task. First Order Features are most prevalent in the RU and LL lobes with 7 occurrences each, totaling 27 across all lobes. Shape Features are relatively rare, with just 4 occurrences across all lobes and appearing mostly in the RM lobe. GLCM features are most common in the LL lobe with 9 occurrences, with a total of 37 across all lobes. GLSZM features are most common in the RM lobe with 9 occurrences, totaling 29 across all lobes. GLDM features are most prevalent in the RL lobe with 6 occurrences.

In our study, three critical features are important in our model’s decision-making process. (1) First Order Features: These features, capturing basic statistical properties of the image intensities within the ROI, were predominantly influential in the RL and RU lobes. Their high frequency of occurrence and significance suggest their utility in capturing intensity variations that are crucial for COPD severity distinction. (2) GLCM Features: Particularly dominant in the LL lobe, these texture features are critical as they capture the spatial relationships between pixel intensities, which are pertinent to understanding tissue heterogeneity in COPD. (3) GLSZM and GLRLM Features: Their uniform presence across several lobes highlights their role in assessing larger area variations and the length of uniform runs in pixel values, respectively, providing key insights into the structural changes within the lung tissues.

In both tasks, the total number of features across all lobes and categories is 104 for the four categories and 132 for the two categories. This data provides valuable insights into the distribution and prevalence of radiomics features across different lung lobes, which could be instrumental in the development and refinement of machine learning models for lung disease diagnosis and severity staging.

## Discussion

In this study, we proposed a novel lobe-based radiomics model for the precise staging of COPD severity using CT images. Our study found that when assessing COPD severity, combining radiomic features from each lung lobe offered a superior overview compared to using features from the entire lung. This method provided a more detailed and localized perspective of COPD severity, highlighting regional variations in disease progression.

Our approach offers many advantages. The localized analysis not only provided a comprehensive understanding of the regional variations in COPD severity but also allowed for a more personalized assessment of disease progression. Additionally, our method facilitated finer biological insights into the differential impact of COPD across the lung lobes. These insights could potentially inform targeted therapeutic strategies in the future. Finally, our predictive models, which combined radiomic features from each lobe, showed superior performance compared to traditional models using features from the entire lung.

The previous studies have focused on using radiomic features derived from the entire lung, as shown in Table 7. Li et al. proposed a method of binary classification of COPD severity with LR and SVM classifiers based on 2D ROI manually marked by doctors to generate 3D VOI to extract image radiomics features. The accuracy was 76.3% [37]. Makimoto et al. [23] and Puchakayala et al. [24] first extracted the features from the whole lung region. Then, binary classification of COPD severity was performed using Linear-SVM and CatBoost. Finally, an AUC was achieved with 78% and 90%, respectively. In the study by Yang and colleagues, the severity of COPD was stratified into four categories. This classification utilized features extracted from the entire lung region [44]. Gonzalez et al. joined four specific CT slices into an image to classify COPD severity using the CNN method, with an accuracy of 51.1% [45]. Sun et al. used three-channel information, including raw CT volumes, segmented lung parenchyma, and emphysema features, as input for 3D ResNet with an accuracy of 76.4% [46].

**Table 7:** Previous studies using radiomic features or deep learning for COPD severity staging

Reference	ROI for feature extraction	Method	Class	ACC (%)	AUC (%)
Li et al. [37]	Randomly select 2D ROI and 3D VOI	LR; SVM	2	76.3%	79.7%
Makimoto et al. [23]	Whole lung	Linear -SVM	2	-	78.0%
Yang et al. [44]	Whole lung	MLP	4	80.0%	94.0%
Puchakayala et al. [24]	Whole lung	CatBoost	2	-	90.0%
Gonzalez et al. [45]	-	CNN	4	51.1%	-
Sun et al. [46]	-	MIL	4	76.4%	91.2%

While these studies have significantly contributed to the field, our approach to analyzing each lung lobe has shown additional benefits. In the experiment, we classify the severity of COPD by extracting the radiomic features of five lung lobes after lung CT segmentation and propose that it is of great significance to classify the severity of COPD based on the combined radiological features of five lung lobes. We also classify COPD severity quadripartite and bipartite based on the radiomic features of whole lung area and single lung lobe of Dataset 1. It is demonstrated that radiomic features extracted from the lung lobe are more specific than those from the whole lung region. The combined radiomic features of all five lung lobes are more complete than the radiomic features of the individual lung lobes, which are more suitable for COPD severity classification. We try to classify the COPD severity of each lung lobe as a separate whole after extracting features from each lung lobe through the trained model. After the segmentation of lung lobes and the extraction of the radiomic features of each lobe, we no longer group the features of each lobe, but carry out feature selection on the features of the five lobes respectively, and then carry out the four-way classification and two-way classification of COPD severity. In addition, we also segment the whole lung area and then extract the radiomic features, and compare the classification results with the combined radiomic features based on 5 lung lobes.

The result shows that radiomic features based on the combination of lung lobes can better represent the details of the lungs, and can be better applied in the classification of COPD severity. The segmentation of the lung into 5 lobes can better capture the CT radiomic features of the lung than simply observing the lung as a whole and also have a better performance in the classification of COPD severity. Because the location of COPD is not fixed, a single lung lobe is not enough to be used as a standard for classification, and the five lung lobes should be evaluated as a whole. In addition, this study demonstrates the significance of the pulmonary lobe in the classification of COPD severity.

Despite these promising findings, our study has certain limitations that warrant mention. First, our sample size is relatively small, limiting the generalizability of our findings. Our study focuses on a single cohort, which may not fully represent the broader COPD patient population. While our model shows improved predictive performance, there’s a need for external validation in larger, diverse cohorts to confirm its clinical utility. Secondly, we utilized spirometry as the reference standard for identifying and staging COPD. However, the performance of this method is suboptimal when categorizing COPD into four severity levels because these categories rely on setting thresholds for spirometry measurements, which operate on a continuous scale. This means that even a slight change in an individual’s pulmonary function may lead to a different COPD classification. By incorporating additional clinical information such as the 6-minute walk distance, body mass index, exacerbation history, and total scores from the St George’s Respiratory Questionnaire, it becomes feasible to enhance the accuracy of GOLD staging.

Given these limitations, future research should aim to validate our findings in larger, more diverse populations. There is also a need to explore the utility of our proposed method in longitudinal studies to assess its predictive performance over time. Furthermore, while our study focused on radiomic features, integrating clinical and genetic data could potentially enhance predictive accuracy. In addition, Multimodal Measures are currently being applied to the diagnosis of a variety of diseases [47]. In the future, we will study the severity classification of COPD by multimodality combining imaging omics features with other factors. Lastly, exploring the use of advanced machine learning and deep learning algorithms could provide additional improvements.

## Conclusions

The study demonstrates that combining radiomic features from five lobes offers a promising approach for staging COPD severity. The lobe-based radiomics features, which are extracted from five lobes in CT images, can provide more localized formation beyond what can be captured by considering the lung as a whole. The proposed method also provides a more granular understanding of disease distribution and severity, potentially improving patient management strategies. Moreover, we aim to refine the radiomics model further, enabling precise COPD staging and providing enhanced personalized support for individual patients in the future.

While the results are encouraging, we acknowledge certain limitations that must be addressed in future work. Firstly, the study’s sample size was restricted, which may limit the generalizability of our findings. Larger, multi-center studies will be essential to validate our approach and ensure its applicability across diverse populations. Additionally, our study focused on only radiomic features, clinical and genetic data have the potential for the prediction of disease progression over time.

## Data Availability

The data will be available upon reasonable request from the corresponding author (Shouliang Qi, qisl@bmie.neu.edu.cn) after approval by the Ethic Committee of the First Affiliated Hospital of Guangzhou Medical University.

## Abbreviations

COPD: Chronic Obstructive Pulmonary Disease
CT: Computed Tomography
GOLD: Global Initiative for Chronic Obstructive Lung Disease
Leaky ReLU: Leaky Rectified Linear Unit
GLCM: Gray Level Co-occurrence Matrix
GLSZM: Gray Level Size Zone Matrix
GLRLM: Gray Level Run Length Matrix
NGTDM: Neighboring Gray Tone Difference Matrix
GLDM: Gray Level Dependence Matrix
LASSO: Least Absolute Shrinkage and Selection Operator
SVM: Support Vector Machine
MSE: Mean Square Error
